# Supplementary material for: Gemcitabine + Cisplatin + S-1 Treatment for Advanced Cholangiocarcinoma: Cost-Effective, with Better Progression-Free Survival Versus Standard Treatment with Gemcitabine + Cisplatin + Durvalumab
Source: Cancers (Basel). 2025 Dec 12;17(24):3971. doi: 10.3390/cancers17243971 (PMC12730319; doi:10.3390/cancers17243971)
Supplement: Supplementary file 1 [file cancers-17-03971-s001.zip › cancers-3992154-supplementary.pdf]

Supplementary Figure S1.

Love plot of standardised mean differences before and after propensity score matching.

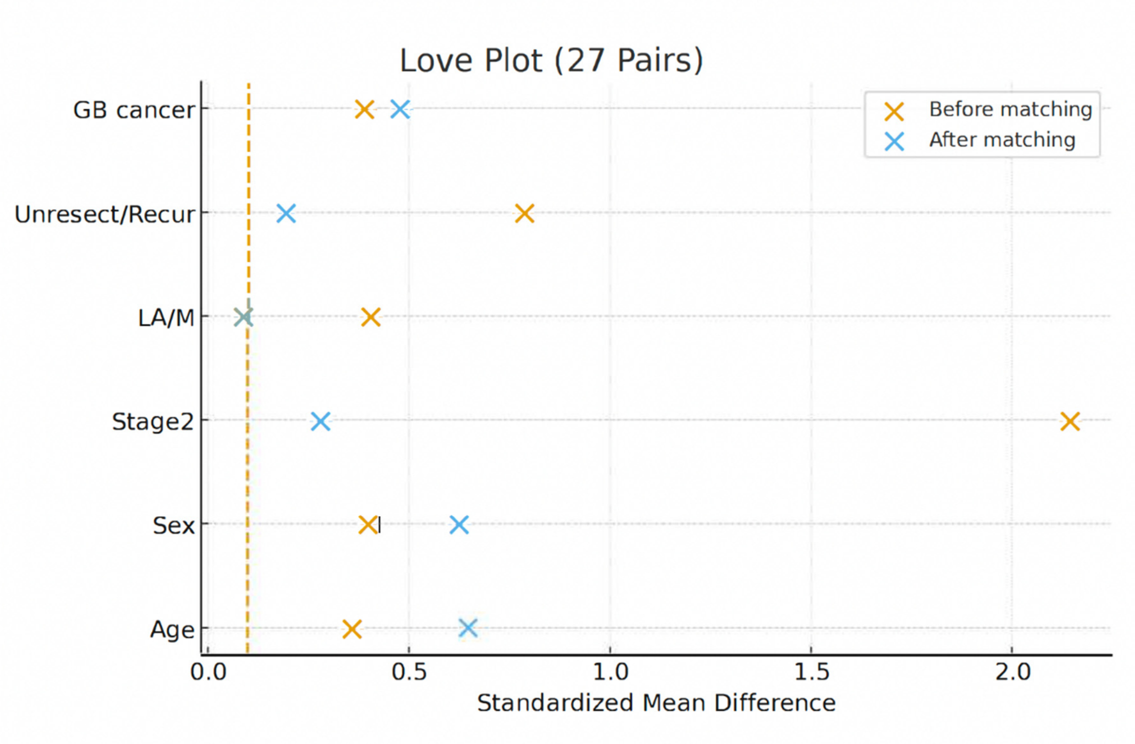

Standardised mean differences for all covariates included in the propensity score model are shown before matching (circles) and after matching (crosses). Covariates included age, sex, stage, extent of disease, disease status, and gallbladder cancer. The reference line indicates standardised mean difference = 0.1. After propensity score matching (n=54; 27 matched pairs), covariate balance improved markedly.

GB: gallbladder, LA/M: locally advanced/metastatic, Unresect/Recur: unresectable/recurrent.

Supplementary Figure S2.
Propensity score distribution after matching.

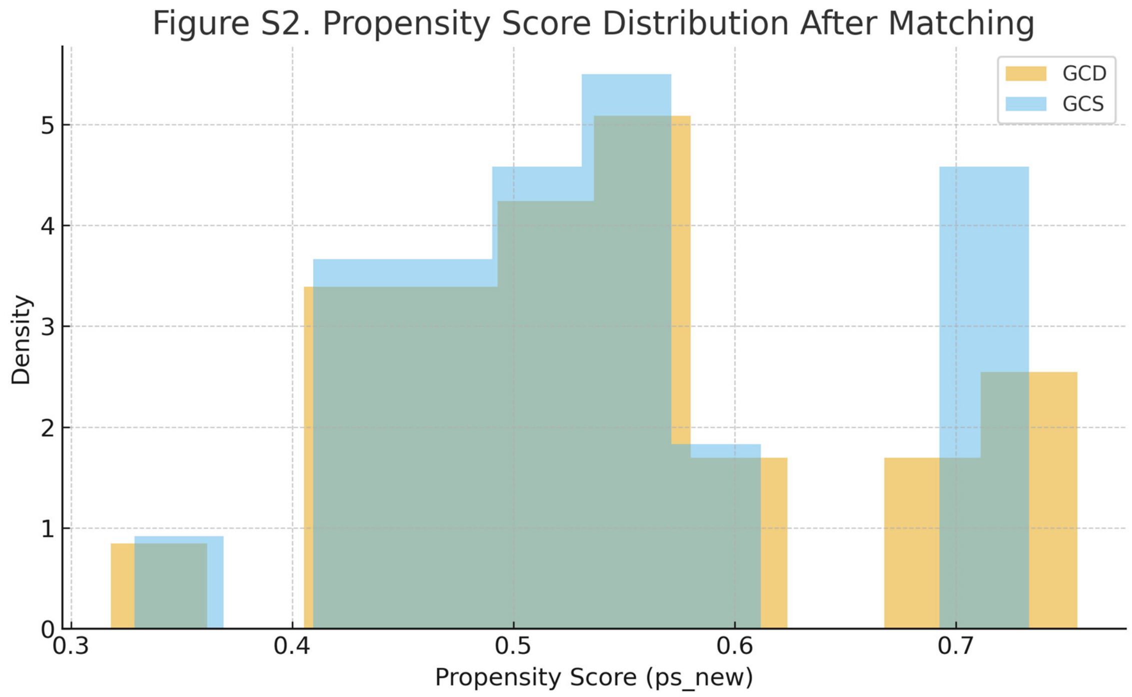

Distribution of propensity scores in the GCS and GCD groups after propensity score matching using the logistic regression–derived propensity score (ps_new). Density histograms illustrate the improved overlap between groups following matching, supporting adequate match quality.

GCS: gemcitabine + cisplatin + S-1, GCD: gemcitabine + cisplatin + durvalumab.

Supplementary Figure S3.

Overall survival by age subgroup (<70/≧70 years).

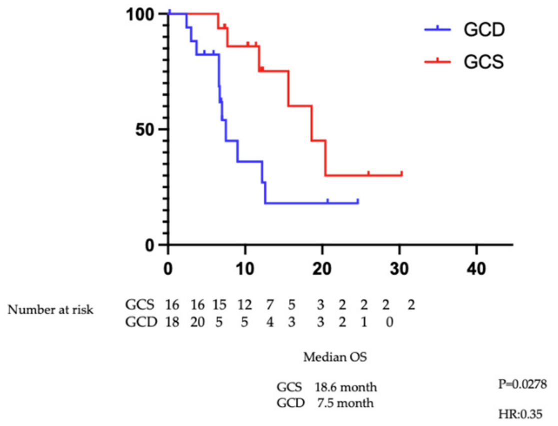

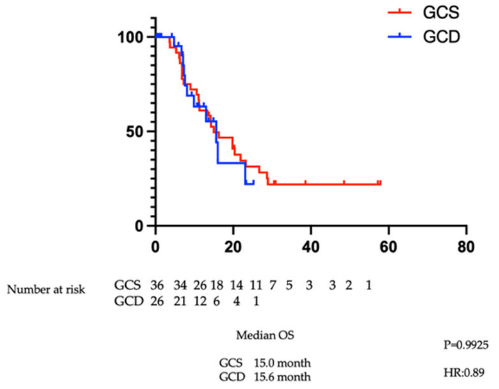

3a 3b

Kaplan–Meier curves for overall survival stratified by age at treatment initiation (<70 versus ≥70 years) in all patients (n=96). Events were defined as death from any cause.

(a) Among patients aged ≥70 years, treatment with GCS was associated with a significantly longer prognosis than GCD.

(b) Among patients aged <70 years, prognosis was comparable between GCS and GCD.

GCS: gemcitabine + cisplatin + S-1, GCD: gemcitabine + cisplatin + durvalumab, OS: overall survival, HR: hazard ratio.

Supplementary Figure S4.
Overall survival stratified by presence or absence of ascites.

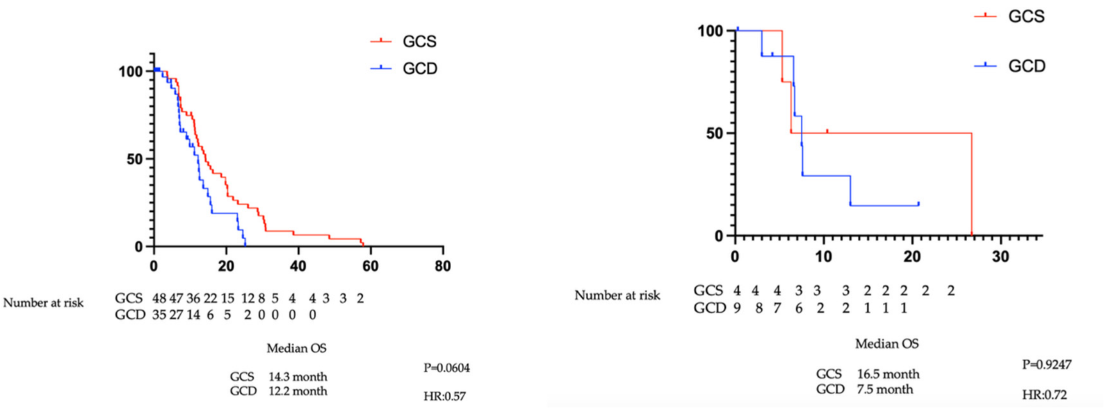

4a **4b**

Kaplan–Meier curves for overall survival stratified by the presence or absence of ascites in all patients (n=96). Events were defined as death from any cause.
(a) In patients without ascites, prognosis was equivalent between GCS and GCD.
(b) In patients with ascites, prognosis was also equivalent between GCS and GCD.
GCS: gemcitabine + cisplatin + S-1, GCD: gemcitabine + cisplatin + durvalumab, OS: overall survival, HR: hazard ratio.

Supplementary Figure S5.

Overall survival stratified by primary tumour site.

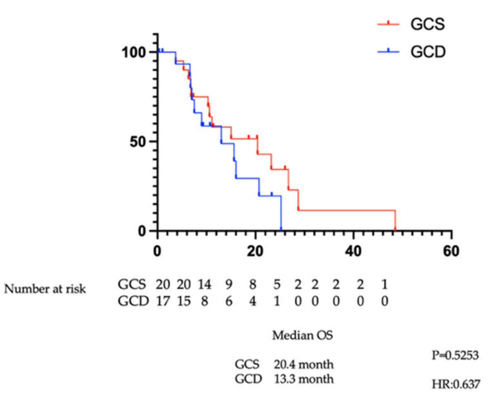

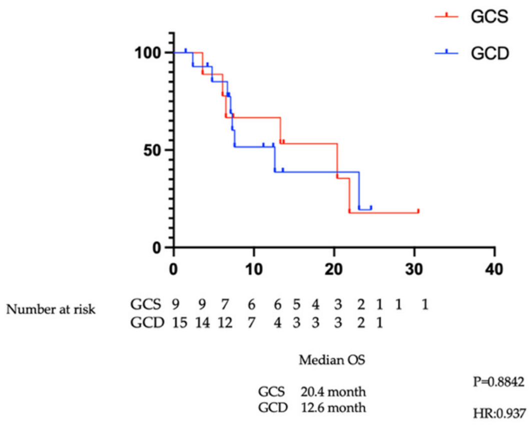

5a **5b**

Kaplan–Meier curves for overall survival stratified by primary tumour site in all patients (n=96). Events were defined as death from any cause.

(a) Among patients with intrahepatic cholangiocarcinoma, prognosis was equivalent between GCS and GCD.

(b) Among patients with gallbladder cancer, prognosis was equivalent between GCS and GCD.

GCS: gemcitabine + cisplatin + S-1, GCD: gemcitabine + cisplatin + durvalumab, OS: overall survival, HR: hazard ratio.

Supplementary Figure S6.

Overall survival stratified by renal function status.

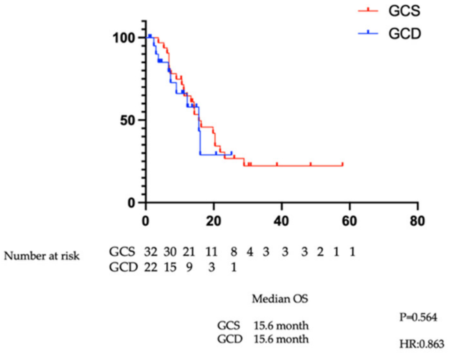

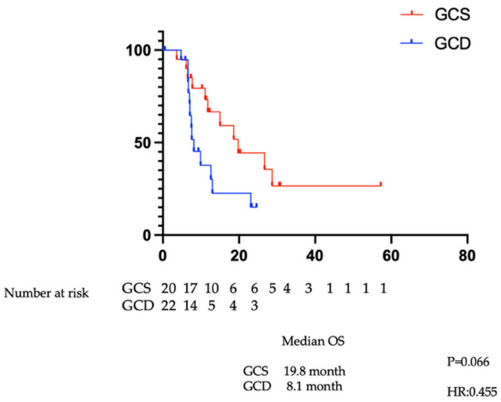

6a **6b**

Kaplan–Meier curves for overall survival stratified by renal function status in all patients (n=96). Events were defined as death from any cause.

(a) In patients with an eGFR of ≥70 mL/min/1.73 m^2, prognosis was equivalent between GCS and GCD.

(b) In patients with an eGFR <70 mL/min/1.73 m^2, treatment with GCS tended to result in a slightly better prognosis than GCD.

eGFR: estimated glomerular filtration rate, GCS: gemcitabine + cisplatin + S-1, GCD: gemcitabine + cisplatin + durvalumab, OS: overall survival, HR: hazard ratio.

Supplementary Table S1.
Baseline characteristics before and after propensity score matching.

Variable	Before matching GCS(n=52)	Before matching GCD(n=44)	SMD	p-value
Age, median (IQR)	68(61.8-73)	65(58.8-70.3)	0.356	0.129
Male sex, n (%)	52.3%	71.2%	-0.392	0.0569
Gallbladder cancer, n (%)	34.1%	17.3%	0.387	0.0585
Metastatic disease(M), n (%)	79.5%	61.5%	0.399	0.0555
Recurrent disease, n(%)	31.8%	3.8%	0.776	0.00025

Variable	After matching GCS(n=52)	After matching GCD(n=44)	SMD	p-value
Age, median (IQR)	68(62.5-73)	63(48-73)	0.649	0.078
Male sex, n (%)	59.3%	29.6%	0.613	0.028
Gallbladder cancer, n (%)	22.2%	44.4%	-0.476	0.083
Metastatic disease(M), n (%)	74.1%	77.8%	-0.085	0.75
Recurrent disease, n(%)	14.8%	22.2%	-0.188	0.48

Baseline characteristics before and after propensity score matching are shown with standardised mean differences and p-values.
GCS: gemcitabine + cisplatin + S-1, GCD: gemcitabine + cisplatin + durvalumab, IQR: interquartile range.
